# Research on virtual reality-based assessment framework and application path in medical education

**DOI:** 10.1371/journal.pone.0310782

**Published:** 2024-11-07

**Authors:** Yue Wang, Yan Li, Chen Chen, Wenli Zhang, Yaping Wang, Kun Sha, Shiyong Wang

**Affiliations:** 1 Faculty of Military Health Service, Naval Medical University, Shanghai, China; 2 International Exchange Center for Military Medicine, Naval Medical University, Shanghai, China; 3 Department of Information, Third Affiliated Hospital of Naval Medical University, Shanghai, China; Guangdong University of Petrochemical Technology, CHINA

## Abstract

While virtual reality(VR) technology enhances learning, it also places new demands on medical learning evaluation. Verifying the occurrence of learning is a primary issue. To design and implement practical and feasible VR-based learning evaluation based on the immersive learning evaluation framework, the Substitution-Augmentation-Modification-Redefinition (SAMR) model, a VR-based learning evaluation framework, was constructed. This framework included competency, learning objectives, assessment tasks, evaluation data, criteria, and feedback. A comprehensive application pathway was developed, utilizing technological integration frameworks. This pathway includes the selection and implementation processes to offer teachers theoretical direction on evaluating medical learning using VR. Finally, this study performed a learning evaluation utilizing VR. The findings revealed that using VR for evaluation can create a deeply engaging and interactive environment. Participants reported feeling a strong sense of being present in the virtual environment and expressed high acceptance and satisfaction with the VR evaluation process. Furthermore, they believed that VR evaluation offers a comprehensive and practical means of assessing cognitive abilities and receiving feedback. These findings establish that VR evaluation optimise learning assessment and showcase the feasibility of the assessment framework and application path.

## Introduction

Medical higher education institutions are increasingly incorporating VR into their teaching methods because of its effectiveness as a learning tool and potential for evaluating learning outcomes [1, 2]. VR has been recognized by the Accreditation Council for Graduate Medical Education (ACGME) as a practical assessment method for evaluating procedural skills and complex clinical tasks [3]. As an effective teaching tool, VR can create an immersive learning environment for experiential learning, inquiry learning, gamified learning, etc., enabling medical students to conduct personalized learning and training in natural, safe, repeatable and automatic feedback scenarios, engaging and motivating students, helping students achieve better learning outcomes, and realizing the transformation from "teacher-centered" to "student-centred" teaching methods [4–6].

Learning assessment collects evidence of medical student learning to measure whether learning is taking place [7] and is one of the core elements of educational activities. However, compared with evaluating learning outcomes, current educational researchers pay more attention to the advantages and values of VR-based teaching and the design and practice of VR-based teaching [8]. Besides, VR-based learning assessments mainly adopt the traditional evaluation method and rarely use the learning behaviour data recorded by the VR system to conduct automatic learning evaluation [9].

To help teachers design and implement practical medical learning evaluation, this paper presents a framework and an application path for VR-based medical learning evaluation based on relevant teaching theories and technological frameworks to measure medical students’ learning outcomes effectively.

## Learning assessment definition

Learning assessment evaluates learning processes and outcomes, directly affecting medical students’ understanding of learning tasks, quality of participation in learning activities, and transfer of learning.

Based on the purpose of evaluation, learning evaluation can be divided into "Assessment of Learning" (AoL), also known as summative assessment, which measures whether medical students have achieved their learning goals, and "Assessment for Learning" (AfL), also known as formative assessment, which helps teachers and medical students collect, interpret and reflect on teaching and learning process data, Assessment as Learning (AaL) stresses that assessment is a form of learning, and is aimed at improving the metacognitive abilities of medical students, helping medical students become active participants in and monitors of their learning through assessment, and promoting natural and continuous learning [10]. Evaluation methods can be divided into non-workplace evaluation based on a traditional examination, evaluation based on a clinical simulation environment, and workplace-based evaluation in a clinical environment [11]. Based on the subject of evaluation can be divided into self-evaluation, peer evaluation, and expert evaluation.

## VR-based learning evaluation framework

Learning evaluation is a fundamental aspect of teaching. There are several models for traditional evaluation, including the Kirkpatrick Model, Anderson Model of Learning Evaluation, Kaufman’s Model of Learning Evaluation, and The Learning-Transfer Evaluation Model [12]. Technology integration frameworks can provide a framework for integrating education and technology, such as the Technological Pedagogical Content Knowledge Framework (TPACK), the Substitution-Augmentation-Modification-Redefinition Model (SAMR), and the Engagement-Enhancement-Extension Model (3E). However, existing learning evaluation models are inadequate in assessing the effectiveness of VR-based learning and the processes involved in learning assessment are distinct from technology integration frameworks [13].

**Fig 1:**
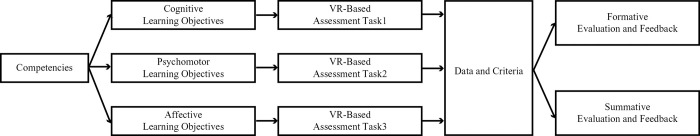
VR-based learning evaluation framework.

To correctly measure whether learning occurred in VR environments, a VR-based learning evaluation framework was constructed based on the Immersive Learning Evaluation Framework [14], SAMR, which includes competencies, learning objectives, assessment tasks, learning data, evaluation criteria, and evaluation feedback, as shown in Fig 1.

**Fig 2:**
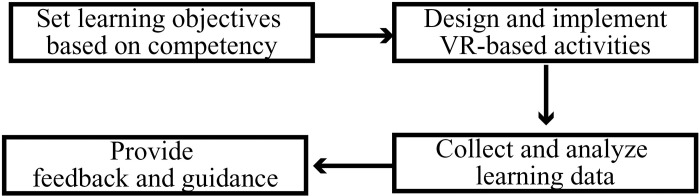
VR-based learning evaluation flowchart.

When conducting VR-based medical learning assessment, teachers need to set learning objectives, including the knowledge, skills, and attitudes that medical students need to master as defined by the competency model, then design VR-based assessment activities, collect learning data, and analyze the learning data based on the assessment criterion, and finally provide targeted learning feedback and guidance, as shown in Fig 2.

### Competencies

Competency-based medical education is a learning outcomes and learner-focused philosophy that emphasizes the critical competencies needed by physicians, ensures that learners acquire theoretical knowledge and practical skills, and assesses medical students using their competencies [15]. Competencies refer to the abilities required of physicians to provide medical care and services. Lee GB et al. define the knowledge, skills, and attitudes needed at each stage of learning, from novice physicians to specialists [16], and serve as the basis for setting learning objectives. ACGME has defined six competencies: patient care, medical knowledge, interpersonal and communication skills, professionalism, practice-based learning and improvement, and systems-based practice [17], as shown in Table 1.

**Table 1:** Competencies for medical student education.

Competencies	Description
**Patient Care**	Provide compassionate, appropriate, and effective patient care to treat health problems and promote health, such as taking medical histories, performing physical examinations, administering diagnostic tests, performing allergy tests, developing patient management plans, etc.
**Medical Knowledge**	Demonstrate knowledge of biological, clinical, and related sciences (e.g., basic immunology, clinical medicine) and be able to apply this knowledge to medical care and services, and so on.
**Interpersonal and Communication Skills**	Communicate effectively with patients, establish and maintain therapeutic relationships with patients’ families, participate in shared decision-making, remove barriers to biased communication, communicate effectively with team members, provide feedback, and so on.
**Professionalism**	Fulfil professional responsibilities and adhere to ethical principles, such as treating patients with respect and compassion, respecting patient privacy and autonomy, and so on.
**Practice-Based Learning and Improvement**	Examine, evaluate and reflect on clinical practice; develop learning plans to improve competence; identify and complete appropriate study tasks to improve clinical practice; and so on.
**Systems-based Practice**	Effectively utilize other resources in the system to provide optimal health care and an awareness of and responsiveness to the larger context and health care system, such as improving patient safety and quality of care by working in interprofessional teams.

### Learning objectives

Medical learning objectives define the expected learning outcomes of medical students, help teachers optimize course design, help medical students focus on learning content, help teachers design and improve course evaluation, and help medical students self-evaluate [18]. There are three domains: cognitive psychomotor, and affective [19], as shown in Table 2. Learning objectives serve as criteria for evaluating learning results and provide a direct basis for teachers to choose appropriate evaluation methods and to design and implement assessment tasks.

**Table 2:** Taxonomy of learning objectives.

Domain	Definition	Category
**Cognitive**	knowledge and the development of intellectual skill	Remembering, Understanding , Applying, Analyzing, Evaluating, Creating
**Affective**	feelings, emotions, and attitudes	Receiving, Responding, Valuing, Organization, Characterization
**Psychomotor**	physical movement, coordination, and use of the motor-skill areas	Perception, Set, Guided response, Mechanism, Comples overt response, Adaptation, origination

### VR-based assessment tasks

The field of medical education has developed medical learning assessment methodologies and tools to assist teachers in the comprehensive evaluation of medical students [20]. However, these traditional assessment methodologies, including practical examination, direct observation, and simulation, lack VR. Compared to traditional evaluations, VR evaluation offers high immersion and strong interaction [21]. When evaluating learning outcomes using VR, teachers can combine one or more VR assessment tasks with traditional ones to provide compelling evidence. Based on the SAMR model, teachers can use VR to replace traditional assessment methods without changing its function, enhancing it by improving its function, modifying it by redesigning tasks, creating new tasks previously unimaginable with traditional assessment methods, and integrating technology into learning assessment, as shown in Table 3.

**Table 3:** SAMR model.

Stage	Description
**Substitution**	Direct replication of traditional evaluation activities, mapping traditional evaluation relationships in VR, providing remote or safety evaluation
**Augmentation**	Optimizing traditional evaluation activities, conducting situational evaluation in VR, and using multiple interactions to conduct multi-sensory participation evaluation.
**Modification**	Re-designing traditional evaluation activities, using VR to collect and analyze physiological data, and conducting a combination of behavioural, physiological and psychological evaluation.
**Redefinition**	Innovating traditional evaluation activities in the virtual medical ecology parallel to the actual medical system, medical students participate in the evaluation as virtual avatars.

#### VR integration mode

At the ‘Substitution’ stage, teachers can digitize traditional assessment tasks to overcome the limitations of time and space. VR provides a safe and immersive method for learning assessment. For example, medical students can interact with virtual patients in virtual scenarios. VR allows for the automatic recording and analysis of learning behaviors, an effective alternative to traditional standardized patients.

Furthermore, at the ‘Augmentation’ stage, VR can present medical scenarios and clinical tasks in three dimensions, simulate the real effects of medical treatment and services, and provide instant feedback to support medical students in collaborative assessment. For instance, the VR surgical evaluation system supports multisensory interactions, feedback, and collaboration, enhancing the evaluation process’s effectiveness.

Subsequently, during the ‘Modification’ stage, the integration of physiological data capturing technologies and VR allows for gathering physiological signal data related to learning. The evaluation approach includes physiological, psychological, and behavioral components by utilizing physiological data, subjective observations made by the evaluator, and objective behavioral data recorded by the system.

Next, at the ‘Redefinition’ stage, with the advancements in artificial intelligence and other technologies, VR has the potential to create a virtual medical ecosystem that runs parallel to the actual healthcare system. VR allows medical students to participate in virtual projects related to medical treatment and service quality enhancement as virtual avatars. Such an approach will overcome the limitations of the natural world and provide innovative testing opportunities for medical students.

#### Technical architecture of the VR evaluation tool

**Fig 3:**
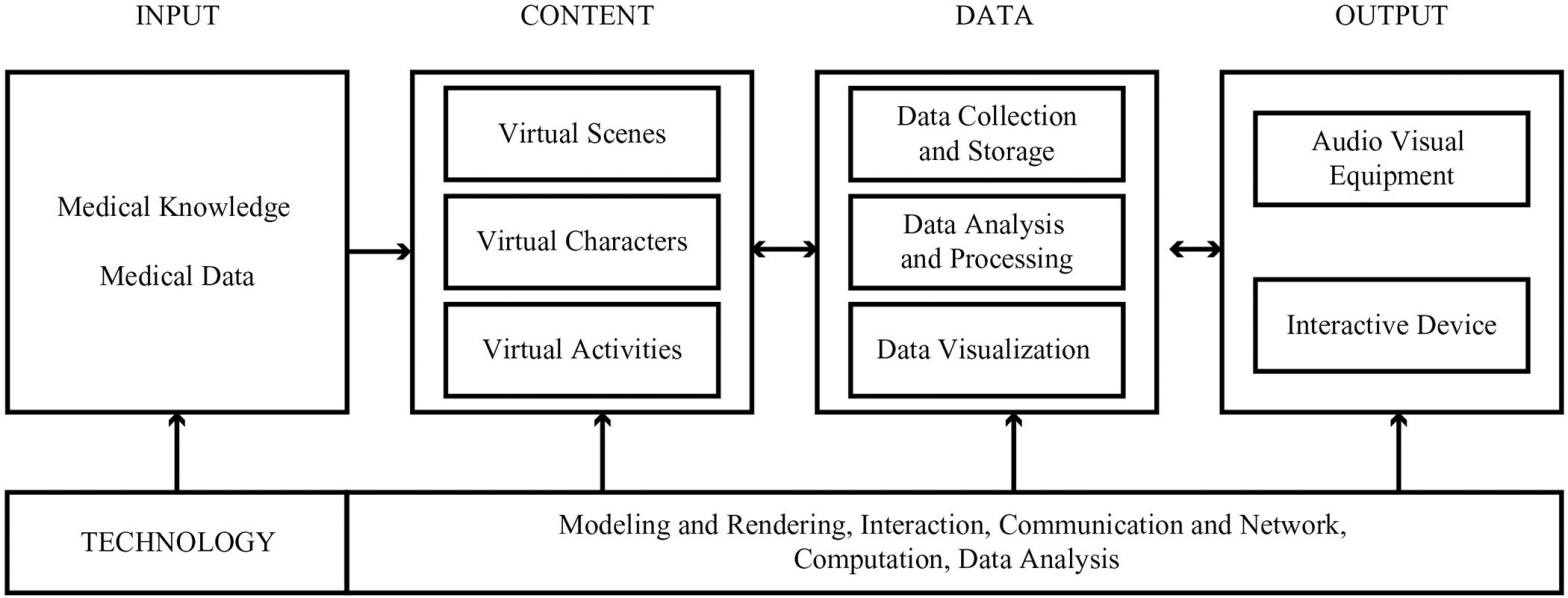
Technical architecture of the VR evaluation tool.

When using VR to assess learning outcomes, teachers must clearly understand the technical architecture of the VR evaluation tool to design, deploy, and implement assessment activities effectively. The VR evaluation tool consisted of five layers: input, content, data, output, and technology support [22–24], as revealed in Fig 3.

The input layer refers to the medical knowledge and data required to create the VR tool, which forms the basis for constructing the evaluation content. The content layer provides the virtual space and interaction necessary for evaluation, including the virtual scenes, characters, and activities. Virtual scenes focus on designing virtual objects, scenes, materials, colors, and other environmental elements, such as classrooms, consultation rooms, and operating rooms, to reproduce realistic environments and simulate human organs. Virtual characters, including virtual patients, healthcare workers, and tutors, interact with medical students during assessment tasks. Virtual activities require solutions for evaluation. The data layer automatically collects, records, and stores the behavioral data of the medical students. It can also process and analyze data and then use the output device to present the multisensory changes generated by the interaction synchronously. Additionally, it can visually display the analysis report and evaluation results.

VR tools require support from various information technologies. Modeling and rendering technology, interaction technology, communication and network technology, computation technology, and data analysis technology are essential for constructing a visualized virtual space and supporting medical students in obtaining multisensory interaction and feedback. These technologies enable stable and low-latency data transmission, scene presentation, instant feedback, user connectivity, and the collection, storage, transmission, and exchange of learning data. Data analysis technology can also effectively analyze behavioral data and provide adaptive assessment tasks.

### Evaluation data and criteria

Conventional assessment instruments can collect qualitative and quantitative data from instructors, students, and peers, such as grades, behaviour logs, performance ratings, etc. VR assessment tools provide a new way of collecting learning data compared to traditional assessment tools [16].

VR tools can use embedded criteria to analyze the real-time performance data of medical students’ learning processes, including completion time, correct/incorrect answers, retry times, access tips, procedure, and operation angle. With external evaluation criteria, teachers and peers can evaluate medical students’ response speed, operation accuracy, and precision using external evaluation metrics, such as on-site observation, VR operation playback, and system logs.

### Evaluation results and feedback

Evaluation results and feedback are effective strategies for promoting teaching and learning and are crucial to learning. While traditional assessments can provide medical students with evaluation and feedback such as test scores, correct or incorrect surgeries, observation logs, and correct surgical procedures, virtual reality assessments can provide formative and summative evaluation and feedback by collecting assessment information as medical students participate in virtual assessment activities [25]. The VR tool can offer immediate feedback to medical students as they learn, helping them understand the gaps between their learning objectives and adjust their learning paths accordingly. Once the learning process is completed, the VR evaluation tool can automatically assess the degree of achievement of the learning objectives and conduct both formative and summative evaluations.

## Application path for VR-based evaluation

Before utilizing VR tools to assess learning outcomes, educators must ensure they are most suitable and effective. This study developed an application path, including a selection path and an implementation path for selecting a VR-based evaluation based on the existing VR learning environment design framework [26, 27].

### Selection path

**Fig 4:**
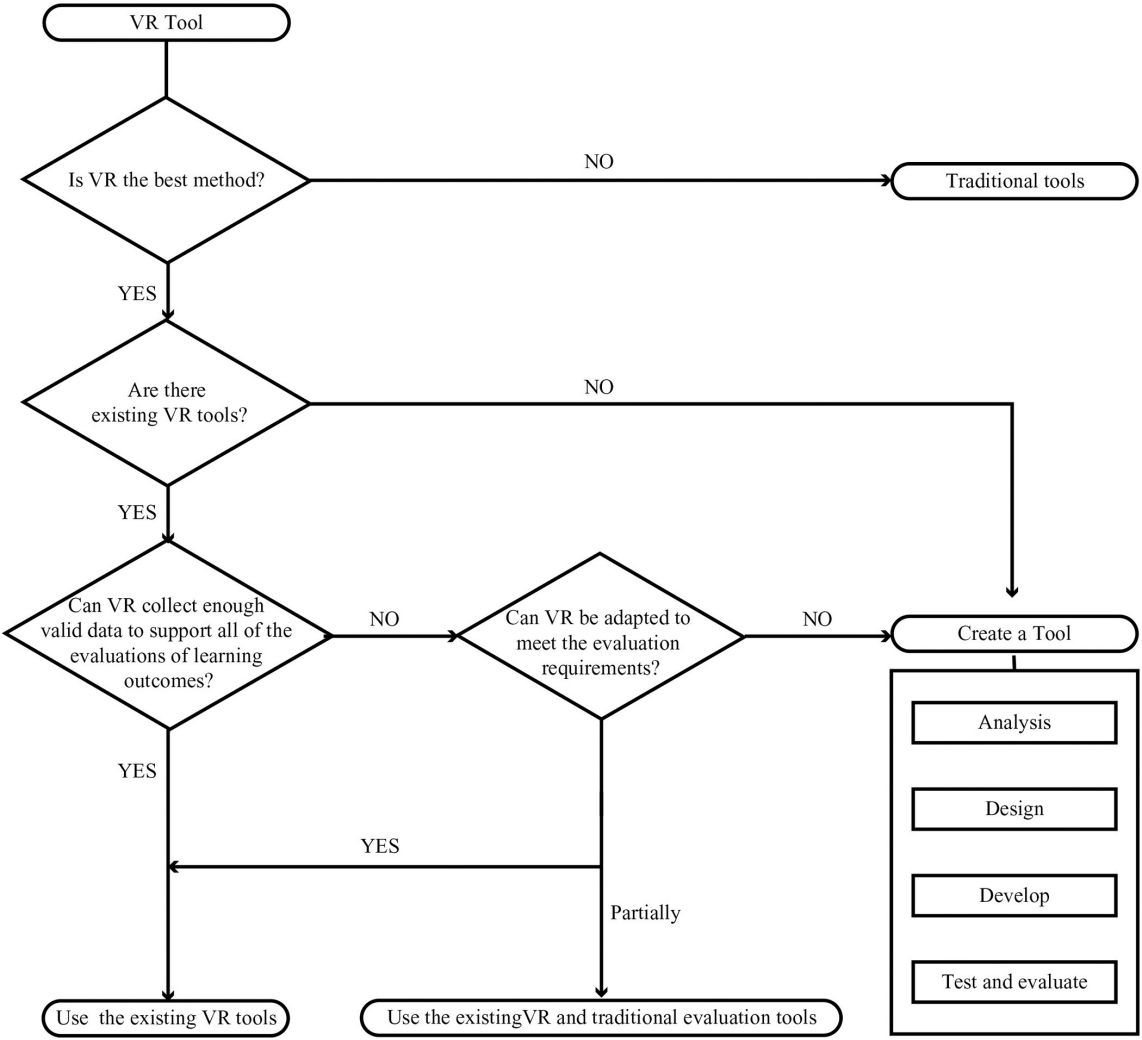
VR tool selection path.

Interdisciplinary teams consisting of teachers, instructional designers, and technologists should analyze the tools based on the purpose and content of the learning assessment. The elements to consider are pedagogical factors such as the virtual environment, teacher-student relationship, software usability, ease of use, and accessibility. After identifying VR as the best approach, the team should research whether current commercial organizations or other colleges and universities have developed tools to meet their needs. They should then determine whether to introduce or design the VR tools, as displayed in Fig 4.

When adopting an existing tool, the team should conduct a trial to determine whether it meets assessment needs. The team can choose VR if the tool can assess all or part of the learning outcomes and the collected data are valid. They can also complement VR with traditional assessment methods to effectively evaluate medical students’ competencies.

Teachers must create and develop new tools if the current VR tools are to be improved to meet evaluation needs. The team should base the tool on the assessment needs when designing it and determine the content, interaction mode (selection, zooming, rotation, movement, and pressing), valid data, evaluation criteria, and device types (immersive VR, semi-immersive VR, and non-immersive VR). They should utilize production tools and engines such as 3Ds Max, Unity, and Unreal Engine to create VR resources and refine VR through iterative testing and evaluation.

### Implementation path

**Fig 5:**
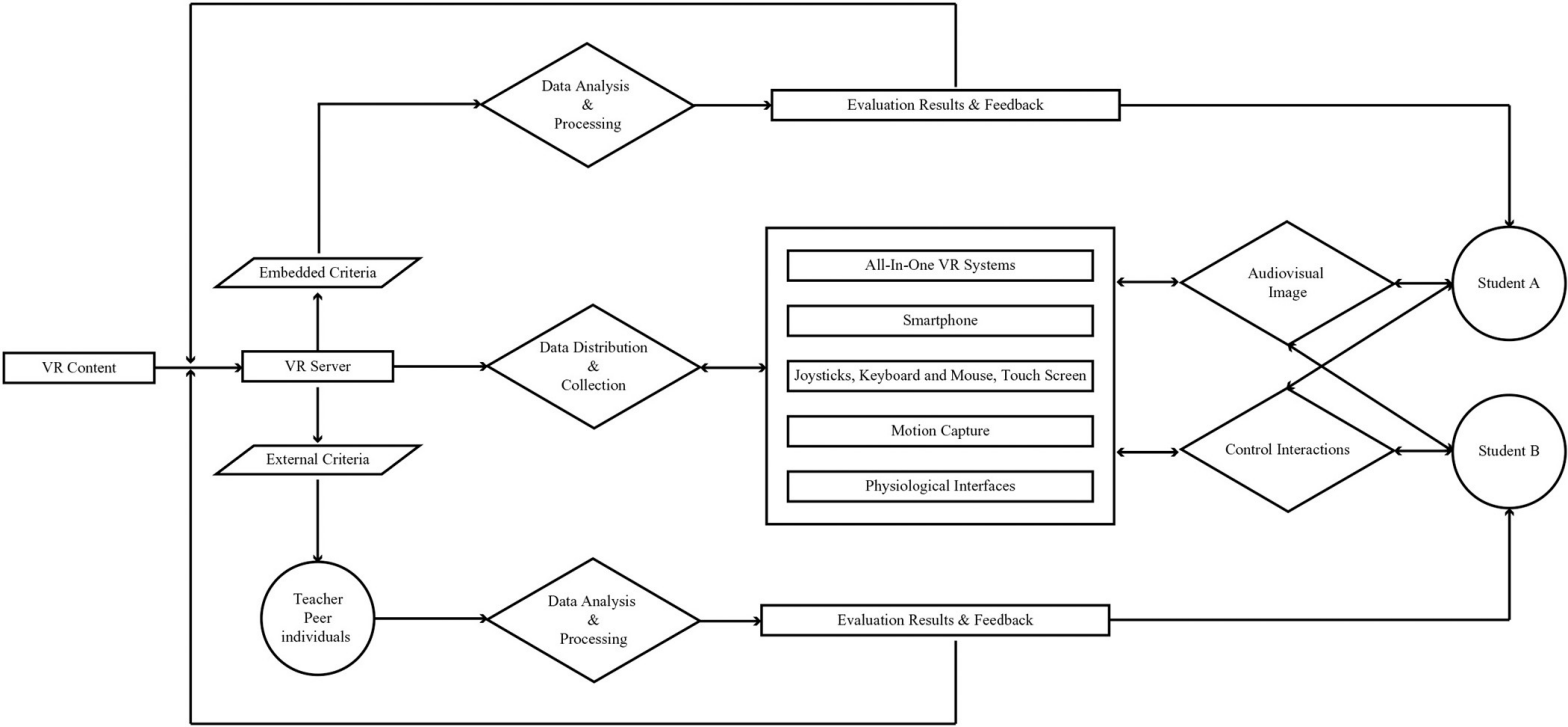
VR-based evaluation implementation path.

This study created a roadmap for VR evaluation implementation based on VR technology architecture [28, 29] to help teachers design VR-based assessment tasks, as demonstrated in Fig 5.

VR resources can be stored, managed, rendered, and executed on local or cloud servers. Teachers can distribute and render virtual resources to VR terminals using wired and wireless networks, cloud computing, edge computing, and other technologies. Medical students utilize external VR headsets, all-in-one VR systems, computer screens, or smartphones to view audio-visual images. Medical students can interact with virtual anatomical structures and patients and complete assessment tasks using joysticks, keyboards and mice, touch screens, motion capture, and physiological interfaces such as brain-computer interfaces. Additionally, they can collaborate with peers in virtual environments to complete the assessment tasks.

VR can record and store personal and collaborative data during the interaction process, including command input data, such as voice, joystick button operation, and screen selection; time data for completing a specific task; body movement data, such as head, eye, and hand movements; and biometric data, such as heart rate, skin conductivity, and EEG. VR can automatically analyze and process data using embedded evaluation criteria to determine medical students’ mastery of medical knowledge, proficiency, precision in surgical skills, and level of empathy. It can provide multichannel real-time feedback using VR terminal equipment and a summative learning evaluation report. Teachers, peers, and individuals can evaluate and provide feedback on VR learning assessment activities based on external criteria for dynamic VR adjustment.

## Research methods

### Participants

For this study, forty sophomore students majoring in clinical medicine were randomly selected from a university, and all students were using immersive VR for the first time. However, some students had used desktop VR devices. These participants engaged in a learning experience using VR equipment, focusing on the content related to appendectomy. Following their engagement with the VR equipment, the participants independently viewed a 360° panoramic video depicting an appendectomy. Subsequently, they participated in a VR-based learning evaluation.

This study does not involve human subject research, animal research, or field research, and all participants were adult college students who were thoroughly informed that the VR-based learning assessment data would be analysed anonymously. Therefore, this study does not require an ethics statement or informed consent.

### Experimental design

The evaluation activity assessed the competency of medical knowledge. The learning outcomes primarily focused on knowledge dimensions, such as the location of Mai’s incision, the method of finding the appendix, and the surgical operation steps. The instructor plans to use virtual resources to simulate appendectomy scenarios and improve the assessment effect. This approach enables students to participate in scenario-based evaluations that engage multiple senses, enhancing traditional assessment methods, as shown in Table 4.

**Table 4:** Evaluate design.

Content	Description
**Competency**	Medical knowledge
**Learning objectives**	**Cognitive domain:** Correctly assess the location of McGregor’s incision Rapidly learn to locate the appendix Properly perform surgical procedures
**Virtual resource**	Customize the assessment of appendectomy to existing resources, reducing the need for precision in the angle of the operation
**VR deployment**	A locally deployed, head-mounted display and controller that is physically connected to the computing devices(HTC Vive)
**Evaluation data and criteria**	Based on embedded criteria, the system automatically evaluates learning behaviour data, including the operation time, position, prompts, and multiple-choice question responses
**Evaluation results and feedback**	**Formative:** The operation time, position, prompts, and multiple-choice question responses **Summative:** The total time to complete the task, the number of prompts requested, the number of trial-and-error attempts

#### VR selection

Immmersive virtual resources are currently available for appendectomy assessments. After considering resource accessibility and deployment costs, the teacher selected a specific resource for the trial. The results indicate that this resource can adequately meet the learning assessment needs. However, it is essential to note that this resource requires high accuracy in surgical tool selection and operating angle. After communication between the teacher and developer, the developer cancels the surgical medical instrument selection task, reducing the precision requirements for operating angles. After being trailed by medical students, the virtual assessment resources began.

#### VR deployment

Teachers deploy virtual resources on localized high-performance computers. Students entered the virtual operating room with a head-mounted display connected to a computer. They interacted with virtual nurses, patients, surgical equipment, multiple-choice questions, and content prompts using joysticks.

#### Evaluation data and criteria

The resource utilizes internal evaluation criteria to automatically record and analyze data on the operation time, position, prompts, and multiple-choice question responses. Medical students will receive immediate feedback on their learning behaviour during evaluation. Upon completion of the assessment, the trainee will receive a summative evaluation that includes the total time to complete the task, the number of prompts requested, the number of trial-and-error attempts made to find the correct operation position, and the results of multiple-choice questions.

#### Survey tools

After completing the learning evaluation activities, the teachers assessed the quality of the VR evaluation through a questionnaire survey. The questionnaire was adapted from the VR learning process questionnaire proposed by Makransky et al [30]. and the evaluation criteria for mobile learning tools proposed by Du Hua et al. [31]. The questionnaire included questions on system characteristics, user experience, and evaluation effects (Refer to S1 Appendix for additional information). The questionnaire used a 5-point Likert scale, and the data were analyzed using SPSS 26.

### Results

Table 5 presents the analysis results of the system characteristics, user experience, and evaluation effects (Refer to S1 Dataset for raw data). The mean of each item was more significant than 4.00, indicating a centralized distribution. Additionally, Cronbach’s coefficients were more significant than 0.7.

**Table 5:** The analysis of system characteristics, user experience, and evaluation effects.

Construct		Mean	SD	Cronbach coefficients
**System Characteristics**	**Representational Fidelity (RF)**	4.53	0.48	0.84
	**Interactivity (I)**	4.50	0.45	0.86
**User Experience**	**Perceived Usefulness (PU)**	4.39	0.50	0.75
	**Perceived ease of use (PE)**	4.30	0.56	0.82
	**Intention to use (IU)**	4.50	0.49	0.85
	**Presence(P)**	4.47	0.42	0.74
	**Satisfaction (S)**	4.46	0.51	0.94
**Evaluation Effect**	**Content(C)**	4.18	0.55	0.77
	**Cognitive Evaluation (CE)**	4.55	0.47	0.93
	**Feedback(F)**	4.43	0.55	0.91

**Fig 6:**
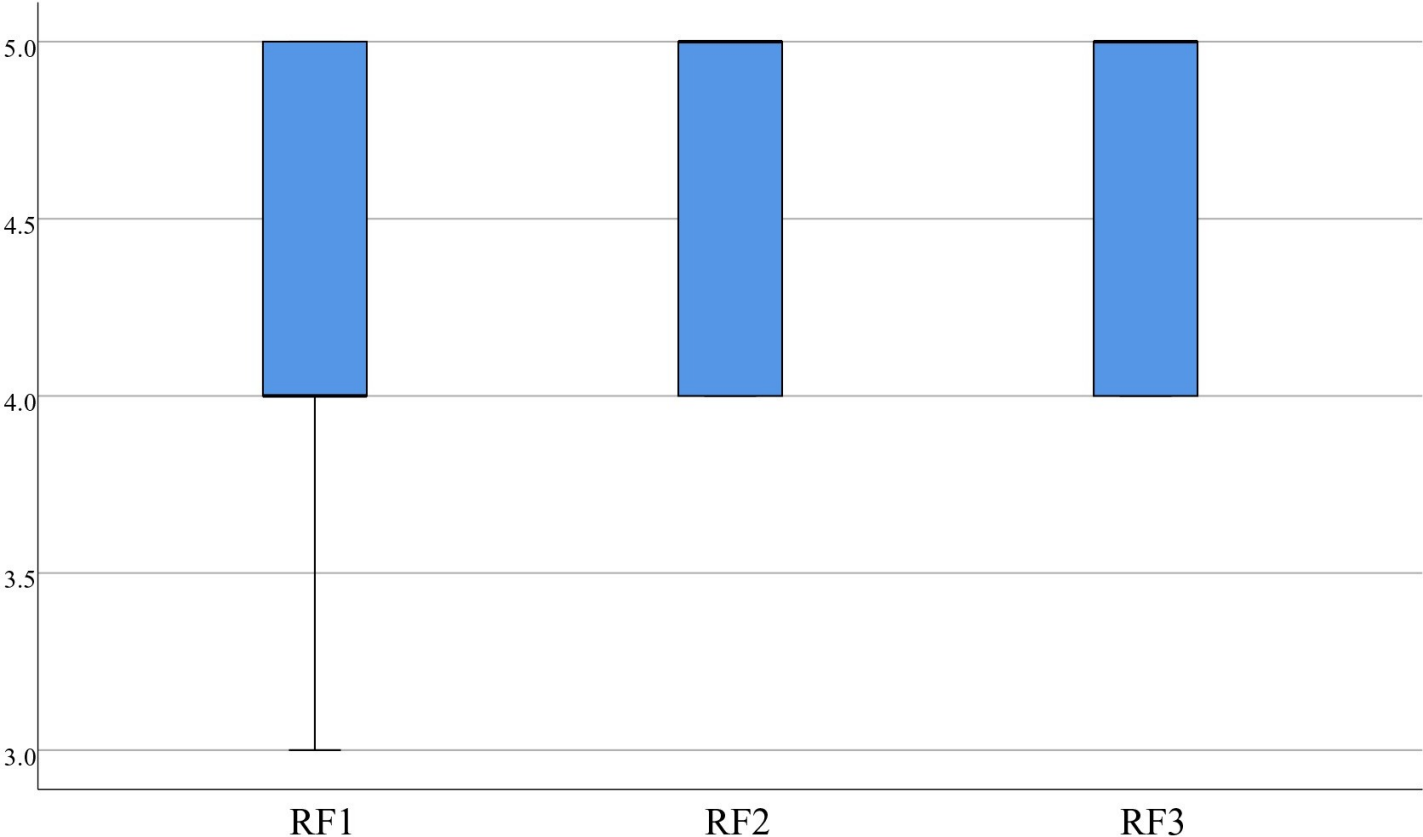
Distribution of RF.

**Fig 7:**
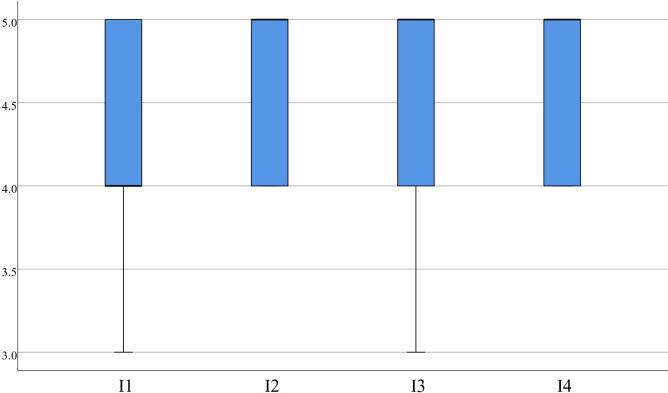
Distribution of I.

**System characteristics.** According to RF statistics, most learners found the virtual scenarios highly realistic and beneficial for their learning assessment experience. Specifically, items 1 (4.30±0.65), 2 (4.63±0.49), and 3 (4.65±0.48) received concentrated scores with a median more significant than or equal to 4.00, as depicted in Fig 6. In the statistics of I, item 1 (4.33±0.57), item 2 (4.53±0.51), item 3 (4.50±0.60), and item 4 (4.65±0.48) demonstrate concentrated scores, with medians greater than or equal to 4.00, which indicates that most learners believe virtual interaction can enhance participant motivation and improve the effectiveness of learning evaluation, as presented in Fig 7.

#### User experience

**Fig 8:**
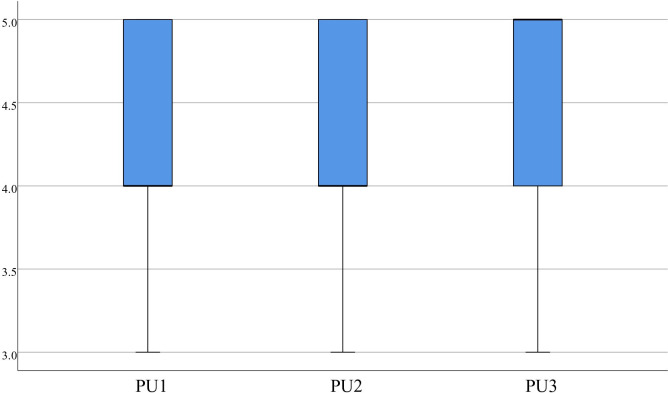
Distribution of PU.

**Fig 9:**
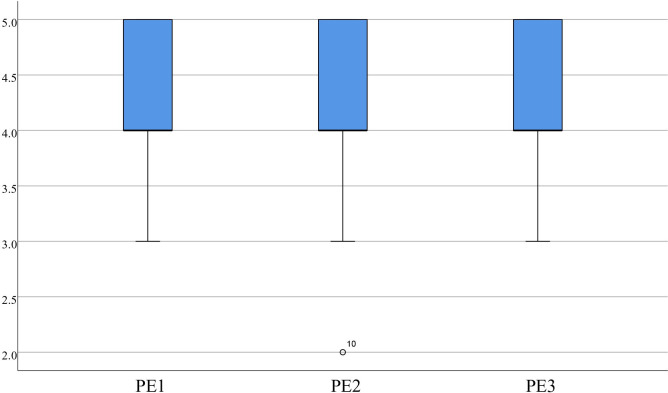
Distribution of PE.

**Fig 10:**
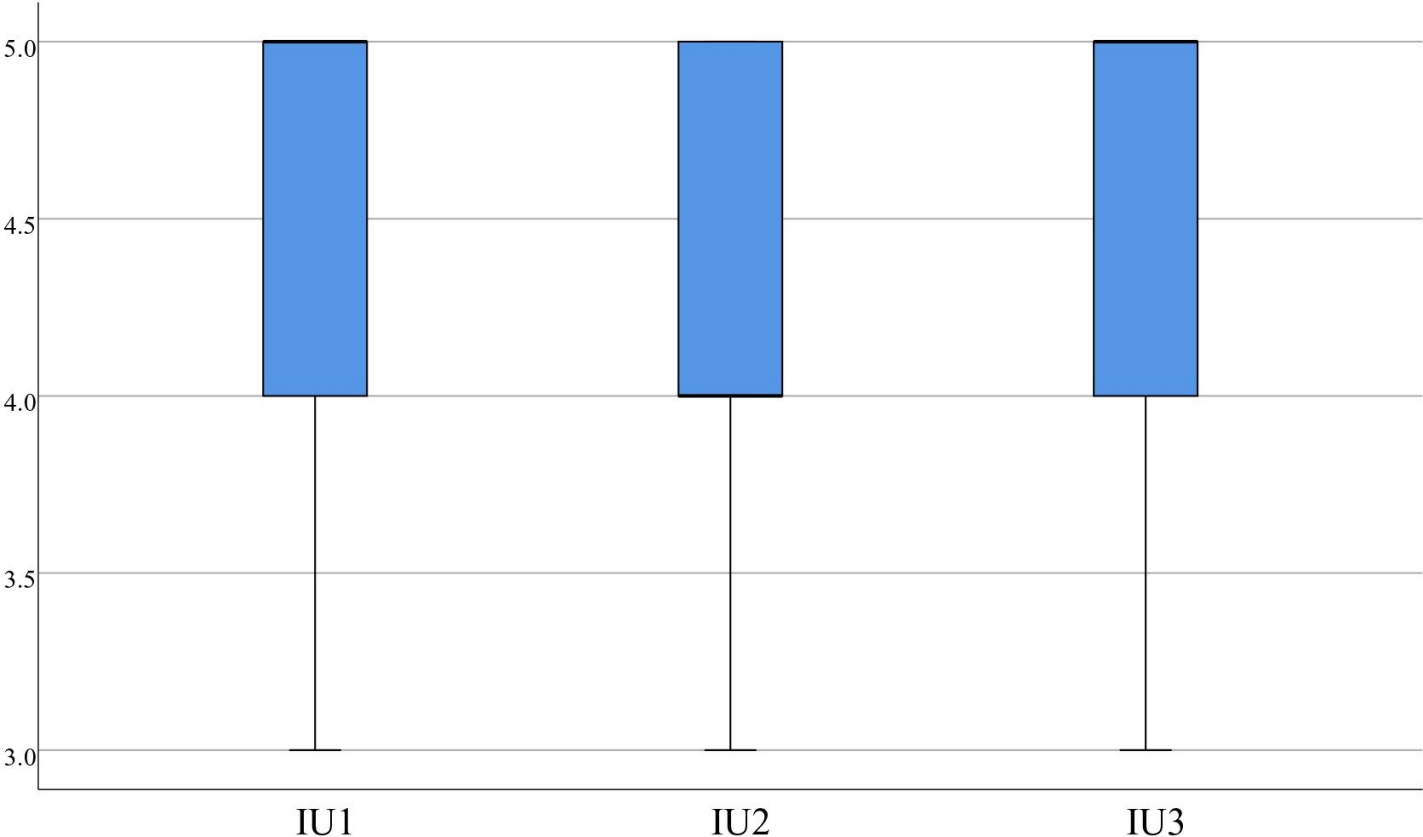
Distribution of IU.

The statistics indicate that for PU, Items 1(4.35±0.53), 2 (4.35±0.70), and 3 (4.48±0.60) had scores concentrated around 4.00 or higher, as displayed in Fig 8. For PE, item 1 (4.28±0.64), item 2 (4.25±0.71), and item 3 (4.38±0.63) also had scores concentrated around 4.00 or higher, as depicted in Fig 9. The statistics on IU also show that item 1 had a score of 4.00 or higher, and the scores for item 1 (4.53±0.55), item 2 (4.40±0.59), and item 3 (4.58±0.55) were concentrated, with the median greater than or equal to 4.00, as presented in Fig 10. It suggests that the medical students found the virtual evaluation resources easy to use and effective and had a high level of acceptance of the VR evaluations. They were also willing to participate in future VR-based learning evaluations.

**Fig 11:**
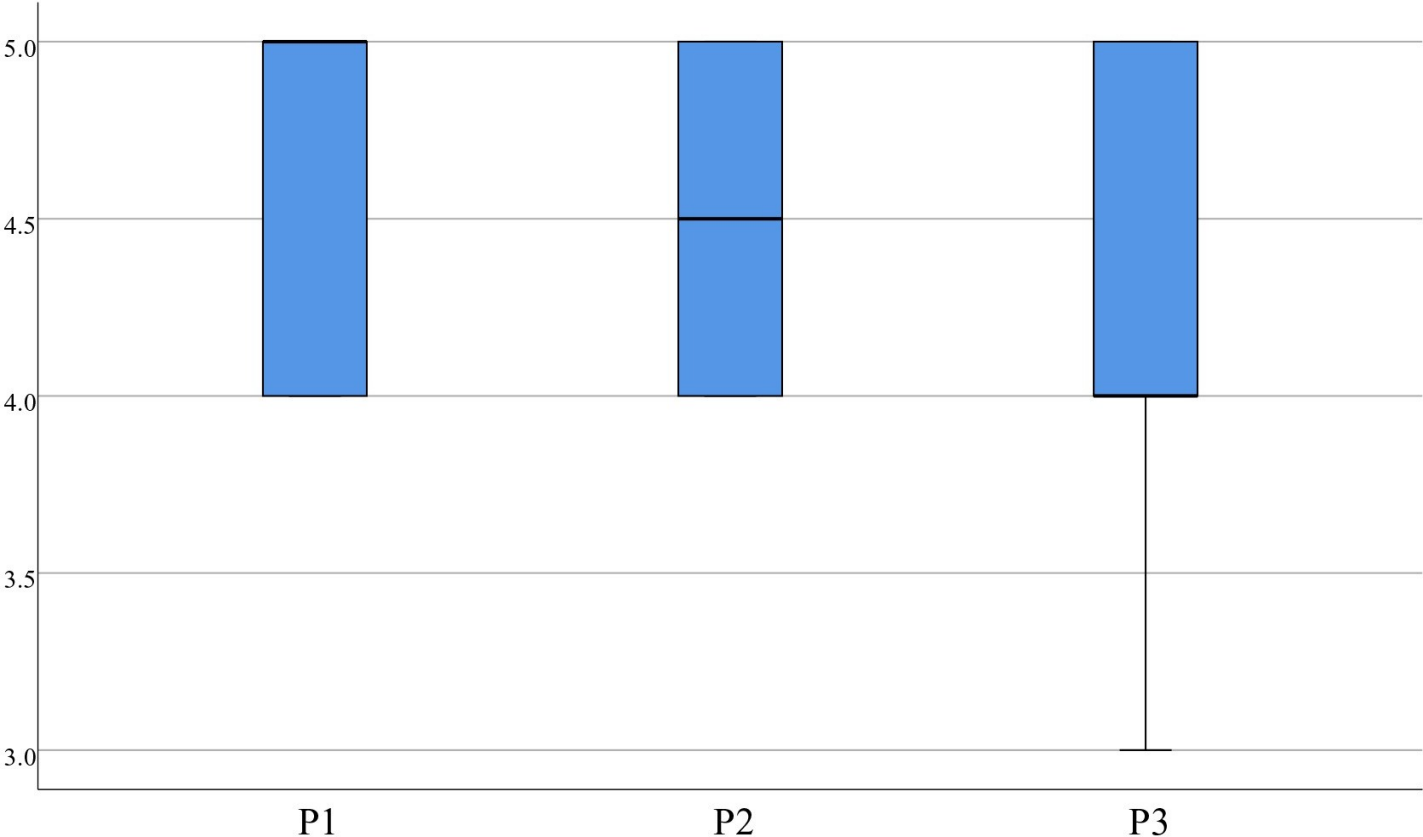
Distribution of P.

**Fig 12:**
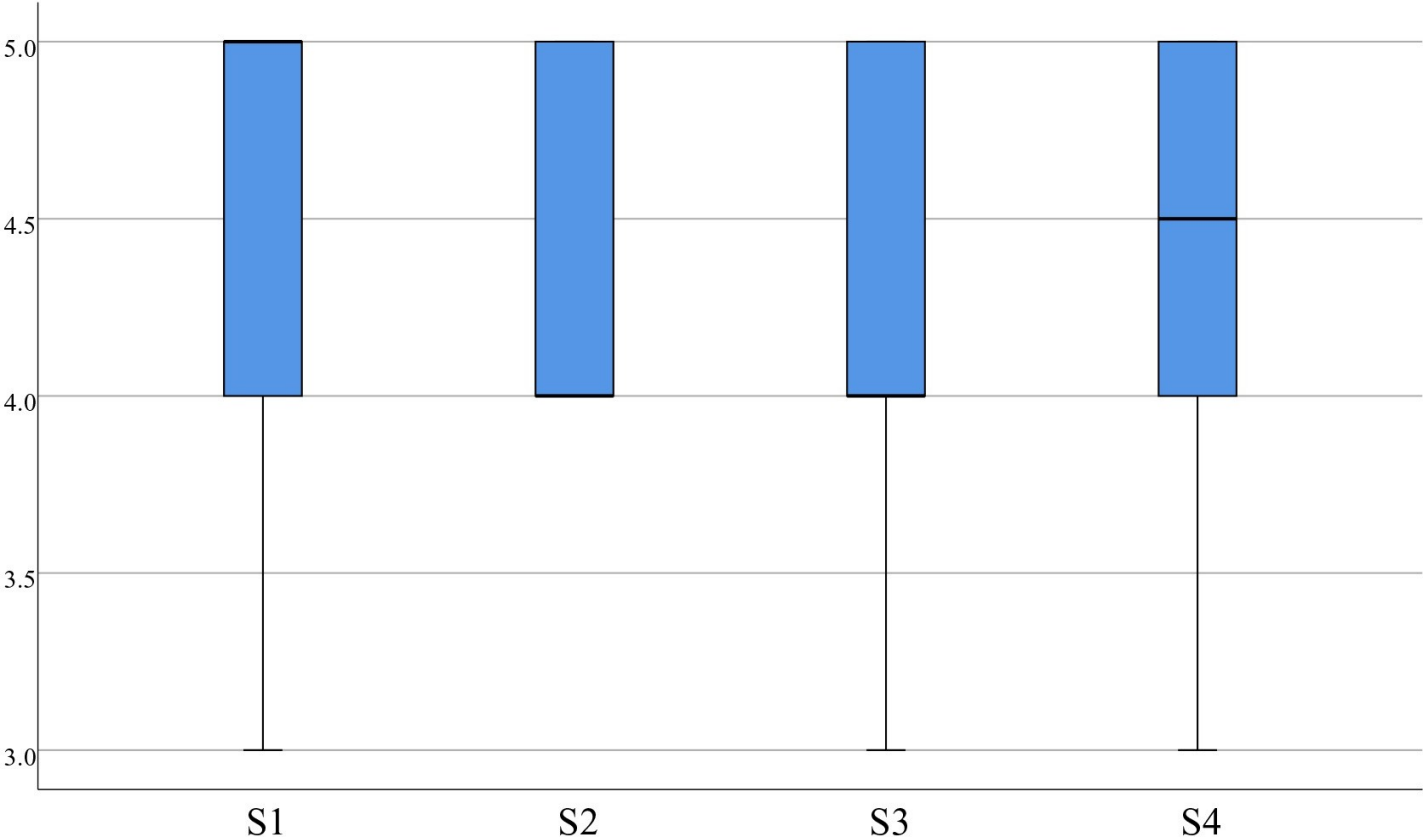
Distribution of S.

In the statistics for sense of P, item 1 (4.53±0.51), item 2 (4.50±0.51), and item 3 (4.38±0.54) had concentrated scores, with a median more significant than or equal to 4.00, which indicates that medical students perceived the virtual evaluation resources as having a strong sense of realism, natural interaction, and a high degree of participation, as displayed in Fig 11. The statistics for S also showed high scores. Item 1 (4.48±0.60), item 2 (4.45±0.50), item 3 (4.45±0.55), and item 4 (4.48±0.55) received high scores, with a median of 4.00 or greater, which indicates that medical students are delighted by the VR-based evaluation methods, as indicated in Fig 12.

#### Evaluation effects

**Fig 13:**
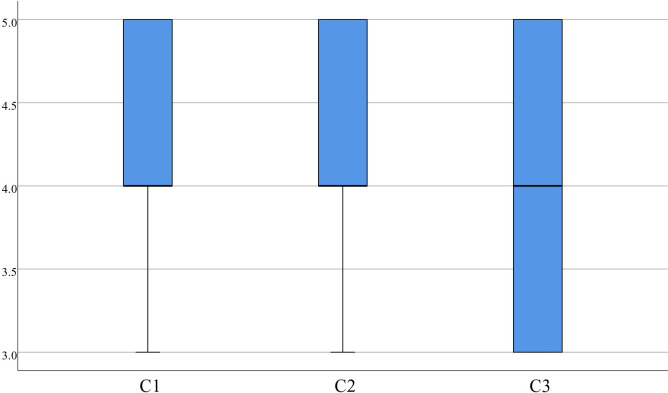
Distribution of C.

Regarding C statistics, the scores for items 1 (4.18±0.68) and 2 (4.35±0.53) are concentrated, with a median of 4.00, which suggests that medical students perceive high consistency between the evaluation content and the learning objectives and that the content comprehensively covers all the learning material. The median score for item 3 (4.00±0.78) was also 4.00, but it was more dispersed than the other items, as displayed in Fig 13. After interviewing medical students who scored 4.00 or less on the third item, teachers found that the high simulation of the surgical field of view provided a realistic surgical experience. Furthermore, virtual surgical operations increase the difficulty of the assessment.

**Fig 14:**
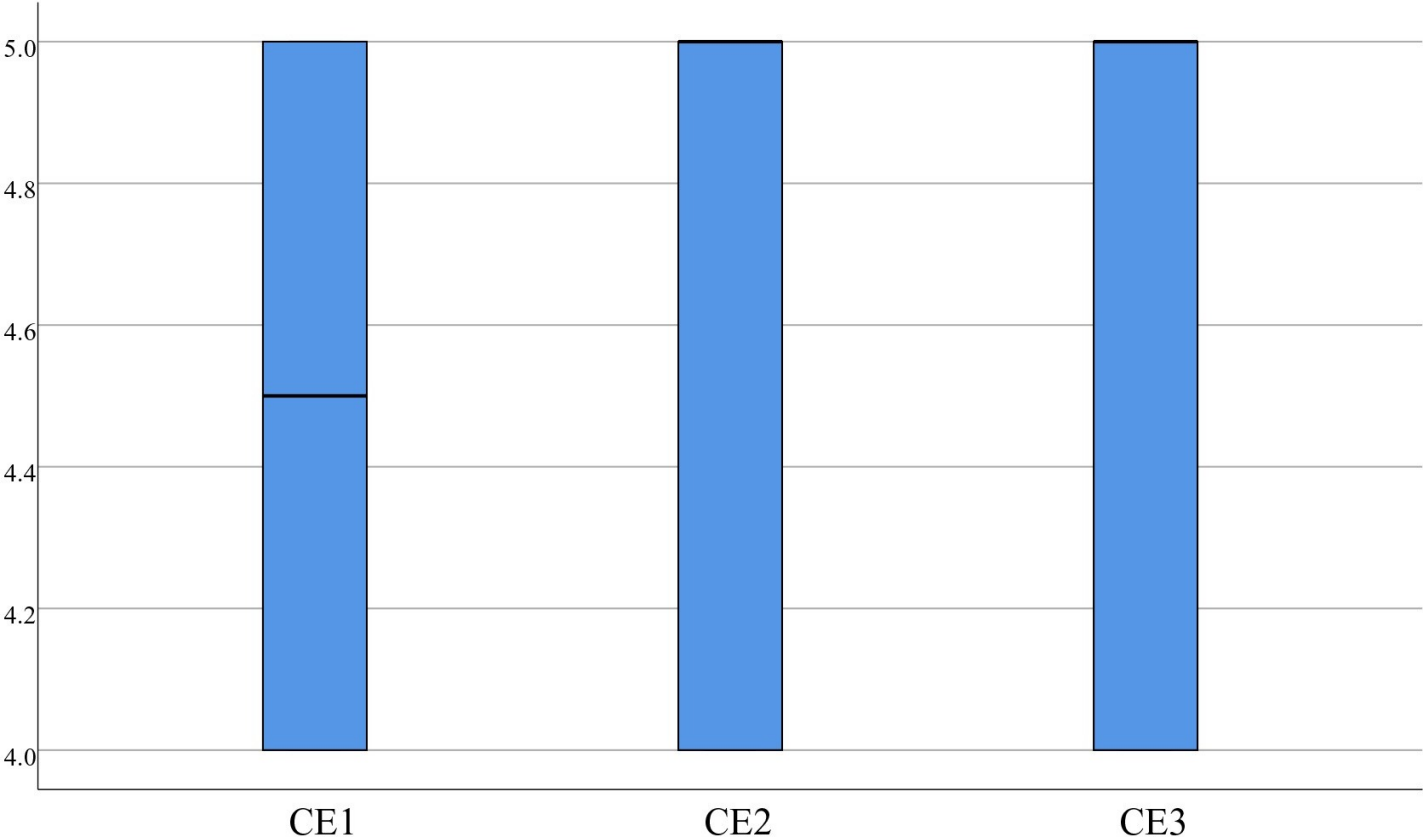
Distribution of CE.

**Fig 15:**
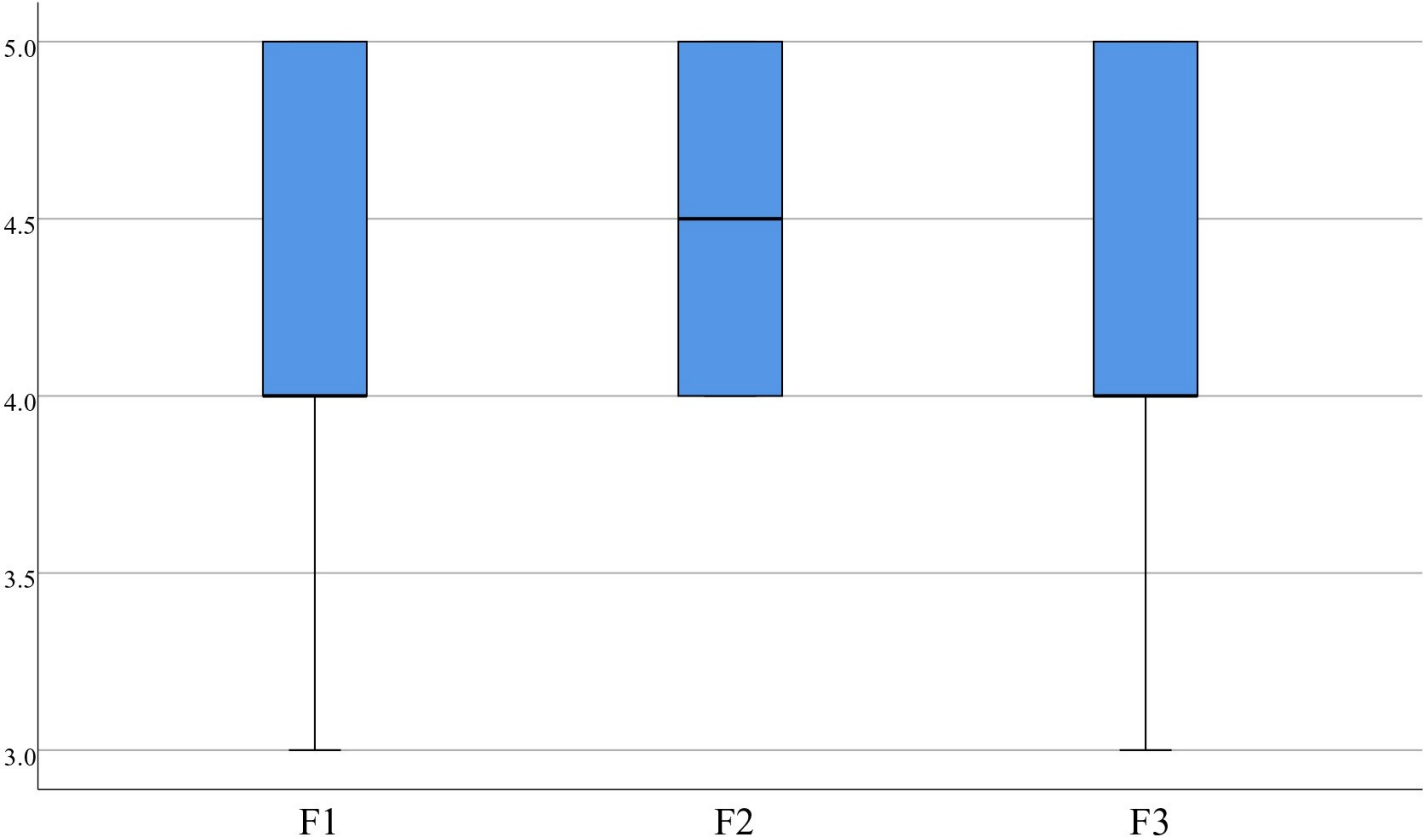
Distribution of F.

Regarding CE statistics, items 1 (4.50±0.51), 2 (4.58±0.50), and 3 (4.58±0.50) had concentrated scores with a median score greater than or equal to 4.00. Medical students believe that VR can effectively evaluate their mastery of knowledge and provide cognitive evaluation, as demonstrated in Fig 14. The F statistics reveal that the scores for items 1 (4.45±0.55), 2 (4.50±0.51), and 3 (4.35±0.70) were concentrated, with a median more significant than or equal to 4.00, which suggests that feedback provided by medical students can assist learners in recognizing gaps in the learning objectives, reflecting on the learning process, and contributing to better learning in the future, as presented in Fig 15.

## Discussion

VR provides an innovative way of evaluating medical education. The framework and application path can help teachers use VR for learning assessment, create learning portraits of medical students, provide learning feedback and guidance, help medical students monitor and control the learning process, promote medical learning, and improve medical students’ competence.

The practical results show that the immersive VR evaluation system has high immersion, can present the 3D evaluation content in high fidelity, and provides effective interaction. The immersive VR evaluation system is practical and easy to use and can provide a natural and immersive environment and a good evaluation experience for medical students. The medical students are delighted with the VR-based learning assessment and are willing to participate in its evaluation activities. The evaluation effect of the immersive VR evaluation system is good; the evaluation content is comprehensive and consistent with the learning outcomes, and the difficulty is appropriate. Learning evaluation activities can provide medical students with a more effective cognitive evaluation, allowing them to display their knowledge and skills better; formative and summative evaluations can help medical students effectively reflect on their learning process and understand the degree of achieving the learning goal.

However, there are still some problems in learning evaluation based on VR. The current immersive VR equipment must be physically connected to the computer terminal, limiting the user’s freedom. It requires much space, and there are problems such as positioning interference in multiple positioning; it uses controllers that provide limited interaction and cannot provide natural perception and interaction; it does not support multi-user collaboration, and medical students can only participate in single evaluation; it lacks resource development capability, and it must rely on society to localize existing resources; most of learning feedback provided is quantitative feedback and personalized learning feedback cannot be provided, which affects medical students’ learning evaluation experience. To further improve the quality of VR-based learning evaluation, it is necessary to use cloud VR devices in the future, introduce wearable devices such as motion capture and haptic feedback gloves, improve the freedom of medical students, realize adequate perception of vital signs, and help medical students personally participate in learning evaluation. Improve teachers’ ability to design, develop and adapt resources, provide more targeted and effective multi-person collaborative evaluation resources for learning evaluation, and improve the effectiveness of learning evaluation; artificial intelligence(AI) will be introduced to realize the in-depth analysis of learning behaviour data and provide personalized learning feedback.

## Conclusion

### Theoretical contribution

The VR-based learning evaluation framework and application path developed in this study can help teachers design practical VR evaluation activities and transition from traditional evaluation methods to situational problem-solving performance evaluations. The outcome-based framework, constructed based on the existing learning evaluation model and technology integration framework, clarifies VR assessment tasks, provides a data-driven evaluation mechanism, and enhances teachers’ comprehension of VR evaluation tools. The application path instructs teachers to select and implement the VR evaluation system, offering precise methodological paths and principles and providing a theoretical basis for carrying out VR-based evaluation activities.

### Practical contributions

The VR-based practice validates and assesses the theoretical model and application pathway, confirms the feasibility of VR-based evaluation, and guides teachers in conducting VR medical learning evaluation. In addition, the "Intelligent Medicine" experience course offered by the Naval Medical University designed and implemented the study and evaluation of hypertension, asthma, allergies, etc., based on the framework and pathway, which further verified the feasibility of the framework and pathway.

Furthermore, an investigation of the evaluation experience of medical students using VR confirmed the effectiveness of the VR tool. The immersive system enhanced students’ learning assessment by providing them with real-world scenarios and interactions, enabling them to experience being fully engaged and actively participating in the evaluation process. The involvement of multiple senses in learning assessments provides students with a more comprehensive cognitive evaluation and feedback.

### Research gaps and future directions

The study’s limitations include the novelty of VR-based learning assessments for most medical students and the potential decrease in the effectiveness of immersive VR evaluations as they become more often used. Furthermore, the impact assessment was investigated through subjective methods. In the future, it will be imperative to evaluate the impact of learning curves, learning retention, and learning transfer on evaluation with those of traditional evaluation tools. Objective learning data should be used to verify whether VR provides the same or better assessment and feedback. Teachers, instructional designers, medical students, and technologists should participate in the iterative process of designing and developing VR-based assessment systems. It is necessary to consider several factors, such as the evaluations’ substance, duration, level of uniformity, ways of natural engagement, and feedback. Moreover, integrating AI, brain-computer interface, biometrics, and other technologies with VR reality can further innovate VR-based learning and assessment, continue to explore the opportunities and challenges of emerging technologies for VR-based learning and assessment in the future, and explore long-term impacts on learning outcomes.

## Supporting information

S1 Appendix VR-based learning assessment experience questionnaire. (DOCX)

S1 Dataset The minimal dataset. (ZIP)
