# Supplementary material for: Research on virtual reality-based assessment framework and application path in medical education
Source: PLoS One. 2024 Nov 7;19(11):e0310782. doi: 10.1371/journal.pone.0310782 (PMC11542797; doi:10.1371/journal.pone.0310782)
Supplement: S1 Appendix — (DOCX) [file pone.0310782.s001.docx]

**S1 Appendix. VR-based Learning Assessment Experience Questionnaire.**

| **Construct** | | **Items** | **Source** |
| --- | --- | --- | --- |
| **System Characteristics** | **RF** | RF1. The realism of the 3-D images motivates me to participate in the learning evaluation. | Adapted from Makransky et al. ^[31]^ and  Du Hua et al. ^[32].^ |
|  |  | RF2. The 3-D images make the learning evaluation more interesting. |  |
|  |  | RF3. The realism of the 3-D enhances my learning evaluation experience. |  |
|  | **I** | I1. The ability to change the view position of the 3-D objects allows me to participate better in the learning evaluation. |  |
|  |  | I2. The ability to change the view position of the 3-D objects makes learning evaluation more motivating and interesting. |  |
|  |  | I3. The virtual environment's ability to manipulate objects (e.g., pick up, cut) makes learning evaluation more motivating and interesting. |  |
|  |  | I4. The ability to manipulate objects in real time enhances the learning evaluation effect. |  |
| **User Experience** | **PU** | PU1. Using this VR as a tool for learning evaluation will promote learning evaluation quality. |  |
|  |  | PU2. Using this VR will enhance the effectiveness of learning evaluation. |  |
|  |  | PU3. This VR is useful in supporting the learning evaluation. |  |
|  | **PE** | PE1. Learning to operate this VR program is easy for me. |  |
|  |  | PE2. It is easy for me to find information with the VR program. |  |
|  |  | PE3. Overall, this VR program is easy to use. |  |
|  | **IU** | IU1. I intend to use VR, assuming I can access them for learning evaluation. |  |
|  |  | IU2. I will use VR frequently in the future. |  |
|  |  | IU3. I want to participate in learning evaluation activities that use VR. |  |
|  | **P** | P1. My interaction with the virtual environment seemed natural. |  |
|  |  | P2. My experiences in the virtual environment seemed consistent with real-world experiences. |  |
|  |  | P3. I was engaged in the virtual environment experience. |  |
|  | **S** | S1. I was satisfied with this VR learning evaluation experience. |  |
|  |  | S2. I was satisfied with the learning evaluation methods in this VR environment. |  |
|  |  | S3. I was satisfied with this VR learning evaluation environment. |  |
|  |  | S4. I was satisfied with the learning evaluation effect of this VR. |  |
| **Evaluation Effect** | **C** | C1. The VR program can test all learning outcomes. |  |
|  |  | C2. The difficulty of the VR program is appropriate. |  |
|  |  | C3. Learning evaluations and learning objectives are closely aligned. |  |
|  | **CE** | CE1. This VR program makes the memory test easier. |  |
|  |  | CE2. This VR program makes the assessment of my understanding easier. |  |
|  |  | CE3. This VR program helps me to reveal better what I have learned. |  |
|  | **F** | F1. VR feedback enables me to reflect on my understanding. |  |
|  |  | F2. VR feedback enables me to reflect on how I learn. |  |
|  |  | F3. VR feedback enables me to become a better learner. |  |
